# Integrating object detection and image segmentation for detecting the tool wear area on stitched image

**DOI:** 10.1038/s41598-021-97610-y

**Published:** 2021-10-07

**Authors:** Wan-Ju Lin, Jian-Wen Chen, Jian-Ping Jhuang, Meng-Shiun Tsai, Che-Lun Hung, Kuan-Ming Li, Hong-Tsu Young

**Affiliations:** 1 Department of Mechanical Engineering, National Taiwan University, Taipei, 106319 Taiwan; 2 Institute of Biomedical Informatics, National Yang Ming Chiao Tung University, Taipei, 11221 Taiwan; 3 Department of Computer Science, National Tsing Hua University, Hsinchu, 300044 Taiwan; 4 Department of Computer Science and Communication Engineering, Providence University, Taichung City, 43301 Taiwan

**Keywords:** Mechanical engineering, Computer science

## Abstract

Flank wear is the most common wear that happens in the end milling process. However, the process of detecting the flank wear is cumbersome. To achieve comprehensively automatic detecting the flank wear area of the spiral end milling cutter, this study proposed a novel flank wear detection method of combining the template matching and deep learning techniques to expand the curved surface images into panorama images, which is more available to detect the flank wear areas without choosing a specific position of cutting tool image. You Only Look Once v4 model was employed to automatically detect the range of cutting tips. Then, popular segmentation models, namely, U-Net, Segnet and Autoencoder were used to extract the areas of the tool flank wear. To evaluate the segmenting performance among these models, U-Net model obtained the best maximum dice coefficient score with 0.93. Moreover, the predicting wear areas of the U-Net model is presented in the trend figure, which can determine the times of the tool change depend on the curve of the tool wear. Overall, the experiments have shown that the proposed methods can effectively extract the tool wear regions of the spiral cutting tool. With the developed system, users can obtain detailed information about the cutting tool before being worn severely to change the cutting tools in advance.

## Introduction

The most important part used in the manufacturing industries consists of machine tools and workpieces. The continuous reaction between the cutting tool and workpiece would generate the phenomenon of tool wear, which is the primary limitation during the machining process. According to the researches^1^, the statistics show that the tool failure would affect approximately 6.8% downtime. With the boosting of the automation industries, the proportion of downtime would increase to 20%^2^. It is important to evaluate the changing time of the cutting tool. When the cutting tool is being worn severely, the cutting tool needs to be changed. However, early replacing the unworn cutting tool will lead to extra costs of waste tools; changing the extremely worn tools too late will take a risk of poor quality of machining products, becoming a waste product. Either the cutting tool is replaced too early or too late increases the waste of resources and industrial costs. A reliable and accurate cutting tool monitoring system could effectively replace the cutting tools, reduce the downtime of tool change, and increase the machining quality. Therefore, evaluating the condition of the machine tool wear in real-time is a critical task in the manufacturing process. To raise productivity, more effective and flexible tool monitoring systems have higher requirements on cutting tools. Recently, monitoring cutting tool technology used to assess the properties of the cutting tool materials, which has been developed to examine how difficult material of cutting tool could be machined^3,4^. Generally, tool wear occurs when the cutting forces and temperature continuously executing on the cutting tool during the machining processing. Hence, the tool wear would further affect the surface quality of the machining workpieces. It is vital to obtain the higher quality of the surface workpiece, which could bring more economical productivity. Reliable tool wear value could be used to effectively schedule the replacement of the cutting tools, and providing the optimum conditions of the machining parameter to avoid tool wear. Therefore, developing the tool wear monitoring system has gained a great deal of emphasis.

There is a considerable body of studies on tool wear monitoring. The technologies of cutting tool monitoring can usually be achieved directly or indirectly based on the cutting tool, workpiece, motor spindles, or machine body. Indirect methods usually measure the signals from various sensors mounting on the machine to indirectly determine the condition of the tool wear, such as cutting force^5,6^, vibration^7,8^, acoustic emissions^9,10^, spindle speed^11,12^. The direct approach of cutting tool monitoring usually uses optical inspection with vision images to directly measure the tool wear geometry in a noncontact process^13–15^. Although a large amount of research work has achieved the successful results in the tool monitoring technique with indirect methods, the challenge of the sensors is generally huge, expensive, and difficult to install, which restricts the convenience of installation. Using the image processing technique can visualize figure out the tool wear condition, which is more available and flexible to confirm the chipped of the cutting tool, the statement of the workpiece, and position of the tool wear area. The earliest image processing technique is based on different grayscale images to set the proper thresholds for selecting the features of tool wear areas^16–19^. However, the traditional methods of image processing highly depend on the parameter setting with complex algorisms and involve expert knowledge to select the appropriate algorism. To minimize the above challenge, lots of researchers have applied artificial intelligence (AI) techniques to overcome the drawbacks of traditional image processing approaches.

With the advancement of technology, the increase of hardware performance and computing speed brings the AI technique explosive growth in the manufacturing industries. AI technology has the characteristics of automatically extracting the important features from raw images and predicting the features based on unlearned images that applying AI technology in the field of image recognition has become a hot topic^20–22^. D. Tabernik et al.^20^ proposed a segmentation-based of deep learning model to detect the surface defect region. Liu et al.^21^ presented a U-shaped deep residual neural network for detecting the quality of conductive particles on the process of TFT-LCD manufacturing. The results gave been shown that the proposed model can detect the particle features from the complex background noise, and obtaining a high recall rate. Lin et al.^22^. utilized CNN model to inspect and localize the defect of LED chip. A large amount of AI-based approaches have been utilized image analysis for tool wear monitoring. Mikołajczyk et al.^23^ implemented a Single Category-Based Classifier neural network to process tool image data and estimated rate of tool wear for cutting edge's flank surface. D’Addona et al.^24^ integrated DNA-based computing (DBC) and artificial neural network (ANN) for managing the tool wear. The experiment demonstrated that the ANN can predict the degree of tool wear from a set of tool wear images processed under a given procedure whereas the DBC can identify the degree of similarity among the processed images. Kilickap et al.^25^ integrated ANN(Artificial Neural Network) and RSM(Response Surface Methodology) models to predict cutting force, surface roughness, and tool wear. The result indicated the proposed method can be used to predict the cutting force and surface roughness effectively. Mikołajczyk et al.^26^ proposed a method based on ANN model for automatic prediction of tool life in turning operations. The results indicated that the combination of image recognition software and ANN modeling could potentially be developed into a useful industrial tool for low cost estimation of tool life in turning operations.

The above reviews of the literature have been shown that the feasibility of applying AI techniques for assessing tool wear. Although lots of researches discuss AI-based methodologies on the topic of tool wear evaluation, very little has considered the deep learning techniques for recognizing the flank wear of spiral tool. Tool wear detection is a task of texture recognition. In recent research, Bergs et al.^27^ utilized the deep learning method to detect the tool wear condition of ball end mill, end mill, drill, and insets based on cutting tool images. The experiments result revealed that the deep learning model of U-net could achieve 0.7 the Intersect over Union (IOU). However, this paper only discussed the detection of single sided cutting tool images. Yet, seldom research has investigated the spiral tool flank wear image based on deep learning technique. It could mainly be attributed to difficulty choose the particular angle of the tool image when measuring the wear of the spiral cutting tool in end milling. Flank wear is the most common wear that happens in the end milling process. It is more important and meaningful to detect the flank wear of the spiral cutting tool. In this paper, deep learning approaches are employed to detect the flank wear for the spiral cutting tool. The current method of detecting spiral cutting tool is to place the cutting tool below the lens and select the specific images of tool wear area perpendicular to the lens for analysis. However, a major challenge in detecting the flank wear area of the spiral cutting tool is that different tool position angles have different values of tool wear area, resulting in difficulty to analyze the curved surface of the spiral cutting tool. To address the above problems, this paper presents an efficient and automatic method for recognizing the tool wear area by using the image stitching method based on pattern match algorithm. The pattern match technique has been widely studied and utilized to solve pattern recognition, which combines multiple images based on point-to-point matching to generate a high resolution image. This method has advantages of high efficiency to achieve wide range of image that several researchers utilized image stitching to merge several scattered images into a complete panorama, which could analyze the features in the panorama image. Gong et al.^28^ developed the template matching algorithm for retina images, which can be used in remote retina health monitoring with affordable imaging devices. Meanwhile, the method solved image quality degradations due to the small field of view (FOV). Generally, a baseline of the cutting tool is used as the criterion of detecting the tool wear area. To effectively find the basic line, this paper proposes a novel detecting method, which stitches the continuous images of the cutting tool images into a panorama image to convert the spiral tool line into a straight line. Furthermore, deep learning techniques are employed to automatically detect the tool wear area. The proposed method is able to capture the tool wear area without the step as selecting the specific area in traditional methods, which can achieve the on-site usage requirements. The developed system could improve the machining efficiency and reduce the frequency of changing the cutting tool.

### Tool wear forecasting approach

**Figure 1:**
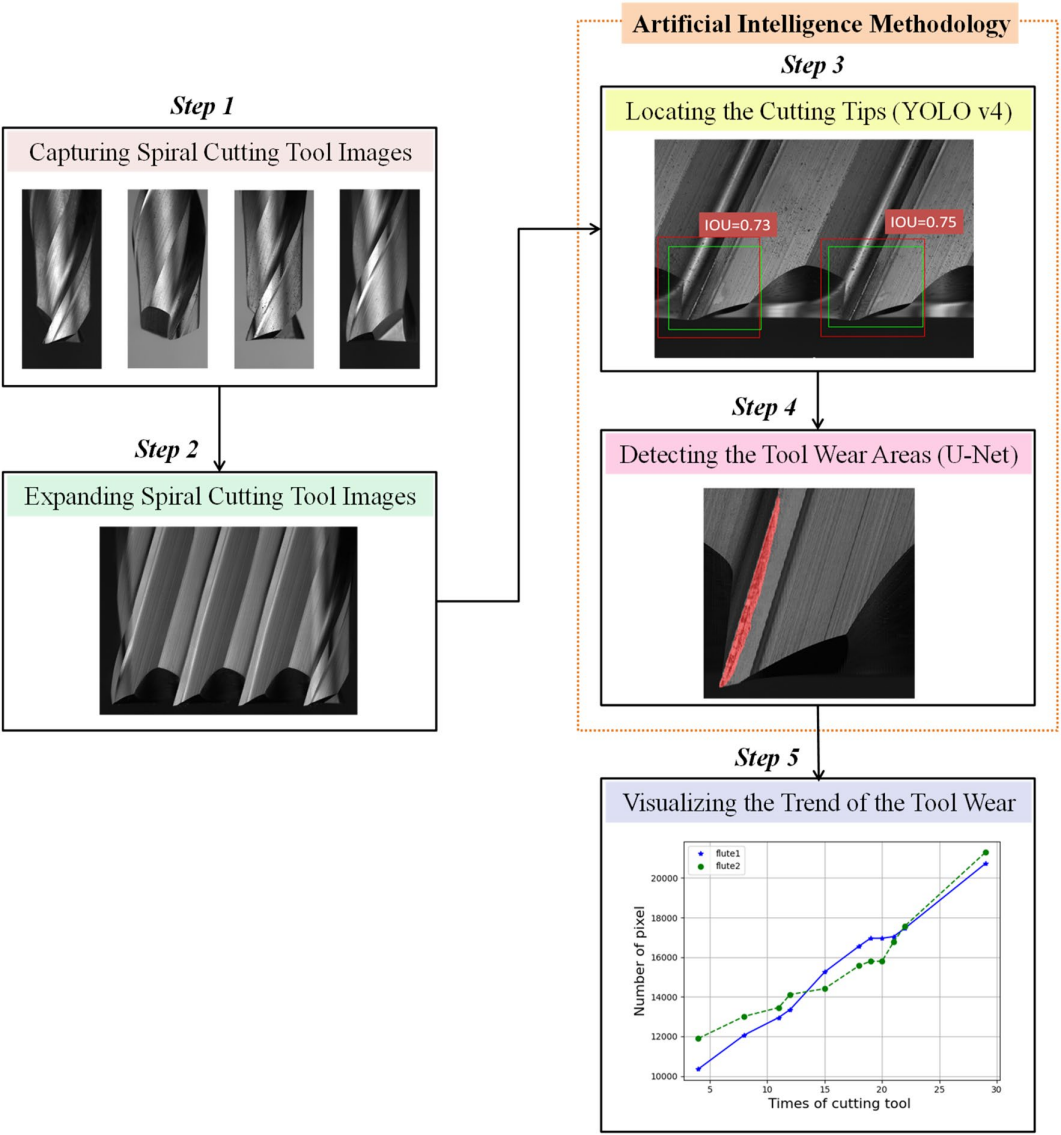
An overview of the developed tool wear detecting system.

An approach of tool wear forecasting is proposed in this study. Image stitching is a mature approach to computer vision, which allows the broader field of vision. Combining image stitching with deep learning techniques can open the new wide of tool wear monitoring. This study aims to use template matching with deep learning techniques to identify and calculate the wear value of the spiral cutting tool. An overview of the developed tool wear detecting system in this study is shown in Fig. 1. The methodology employed in this study mainly contains five stages. Firstly, the tool wear images of the spiral cutting tools were collected for analysis. In the second step, using template matching of image stitching technique to expand the tool images into panorama images. To detect the region of tool wear area more efficiently, deep learning-based object detection and segmentation techniques, instead of traditional computer vision methods, automatically identify the texture of the panorama tool wear images. In the third step, the YOLOv4 model was used for automatically detecting the range of cutting tips based on the panorama images. U-Net was utilized to identify the tool wear texture based on the cutting tips in the fourth stage. And in the final step, the trend of the tool wear areas was visualizing to evaluate the condition of the tool wear in real-time. The employed methods are described in more detail in the following sections.

### Template matching

Template matching is a typical method of image stitching algorithm, which uses a simple machine vision approach to identify the expected template^29–31^. The template matching method is utilized to recognize the locations and characteristics of the reference image from the inspected images. The procedure of template matching mainly included two steps. Firstly, an initial image of the spiral cutting tool is selected as the reference image. Secondly, template comparison is used to extract the features of the overlapping areas by sliding scanning. And then the best correlation area is calculated with the highest similarity of the overlapping area based on the predefined template. In the template matching process, a metric of Normalized Cross Correlation (NCC) value is the metric for matching performance between the template and inspected image^32,33^. The equation of the NCC algorithm is described as follows:

1 $${\text{r}}\left( {{\text{x}},{\text{y}}} \right) = \frac{{\mathop \sum \nolimits_{i = - m/2}^{m/2} \mathop \sum \nolimits_{j = - n/2}^{n/2} \left[ {s\left( {x + i,y + i} \right)*t\left( {i,j} \right)} \right] - m*n*\mu_{s} *\mu_{t} }}{{\sqrt {\left( {\mathop \sum \nolimits_{i = - m/2}^{m/2} \mathop \sum \nolimits_{j = - n/2}^{n/2} s^{2} \left( {x + i,y + j} \right) - m*n*\mu_{s}^{2} } \right)*\left( {\mathop \sum \nolimits_{i = - m/2}^{m/2} \mathop \sum \nolimits_{j = - n/2}^{n/2} t^{2} \left( {i,j} \right) - m*n*\mu_{t}^{2} } \right)} }}$$

where $\mathrm{t}\left(i,j\right)$ represents the template image on the $\left(i,j\right)$ plane, $\mathrm{s}\left(i,j\right)$ is the inspected image on the $\left(i,j\right)$ plane, $m*n$ means the image size, ${\mu }_{s}$ is the mean gray value of the inspected image, and ${\mu }_{t}$ is the mean gray value of the template image. The mean gray value of inspected image and template image are defined as follows:

2 $$\mu_{t} = \frac{1}{m*n}\mathop \sum \limits_{i = - m/2}^{m/2} \mathop \sum \limits_{j = - n/2}^{n/2} t\left( {i,j} \right)$$

3 $$\mu_{s} = \frac{1}{{m{*}n}}\mathop \sum \limits_{i = - m/2}^{m/2} \mathop \sum \limits_{j = - n/2}^{n/2} s\left( {{\text{x}} + i,{\text{y}} + j} \right)$$

### You Only Look Once v4 (YOLO v4)

**Figure 2:**
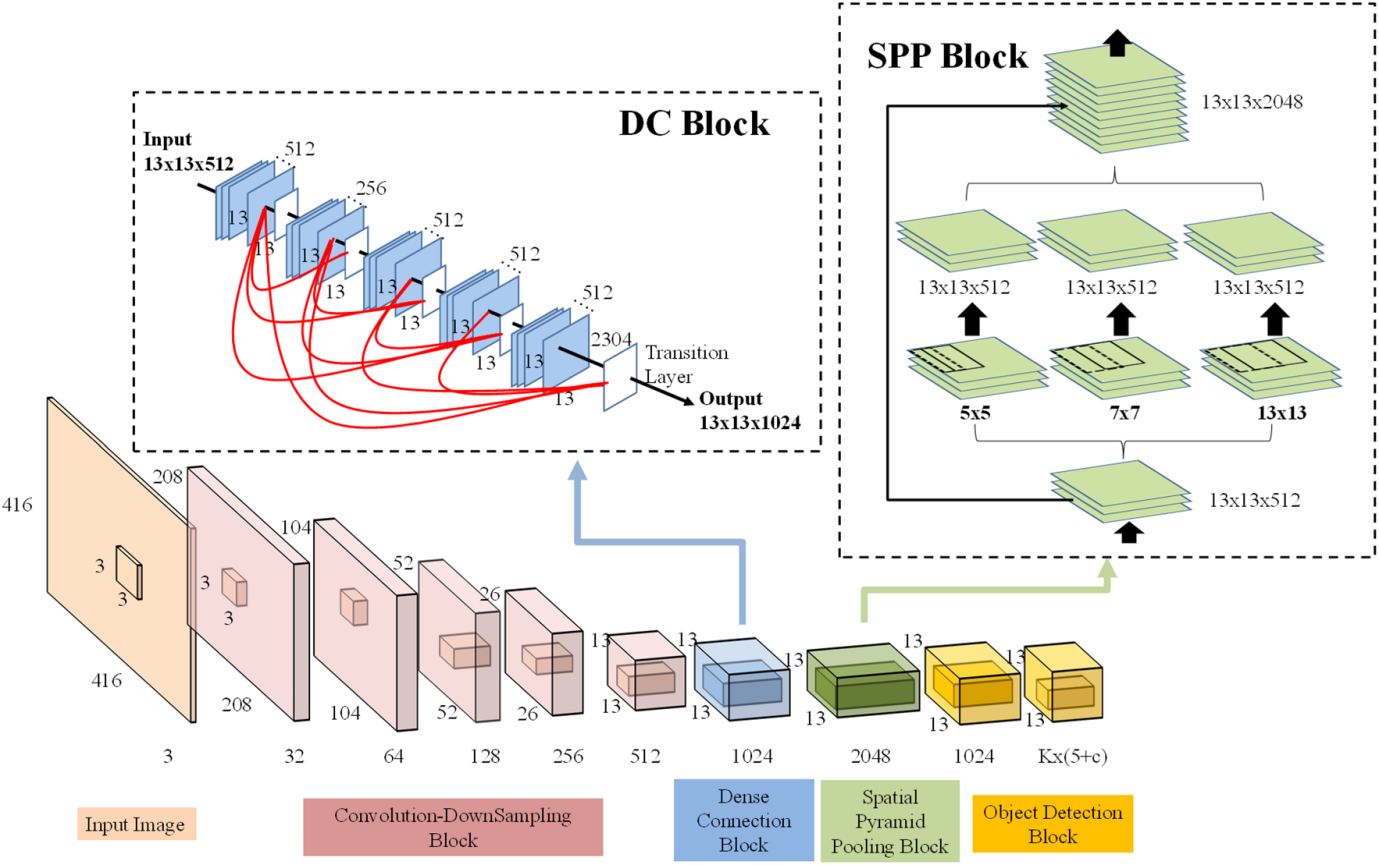
The structure of YOLO v4^34^.

In this study, owing to the larger of the panorama images, the YOLO v4 model is employed to automatically detect the range of cutting tips based on the panorama images, which makes the tasks of segmenting the tool wear texture more efficient. YOLO (You Only Look Once)^34^ is a classical object detection model with the one-stage framework. CNN model can be trained to predict the object with multiple positions and categories at once. The state-of-the-art of YOLO v4 is proposed recently, which can achieve high accuracy in real-time^35,36^. The architecture of YOLO v4 consists of three elements, namely, backbone, neck, and head. The backbone of the YOLO v4 is using the architecture of Darknet53 and Cross Stage Partial Network (CSPNet), named CSPDarknet53 to train the object detection model. Because CSPDarknet53 can achieve high detection accuracy that CSPDarknet53 is utilized to be the backbone of the YOLO v4 model. The neck of the YOLO v4 model is integrated SPP (Spatial Pyramid Pooling) and PANet (Path Aggregation Network) to have a better feature fusion for combining feature maps with different scales. Moreover, the head of the YOLO v4 model is used YOLOv3 head The YOLO v4 model can not only improve the problem of computation time but also enhance the accuracy of model recognition with the above elements. The structure of YOLO v4 is shown as Fig. 2.

### U-Net

**Figure 3:**
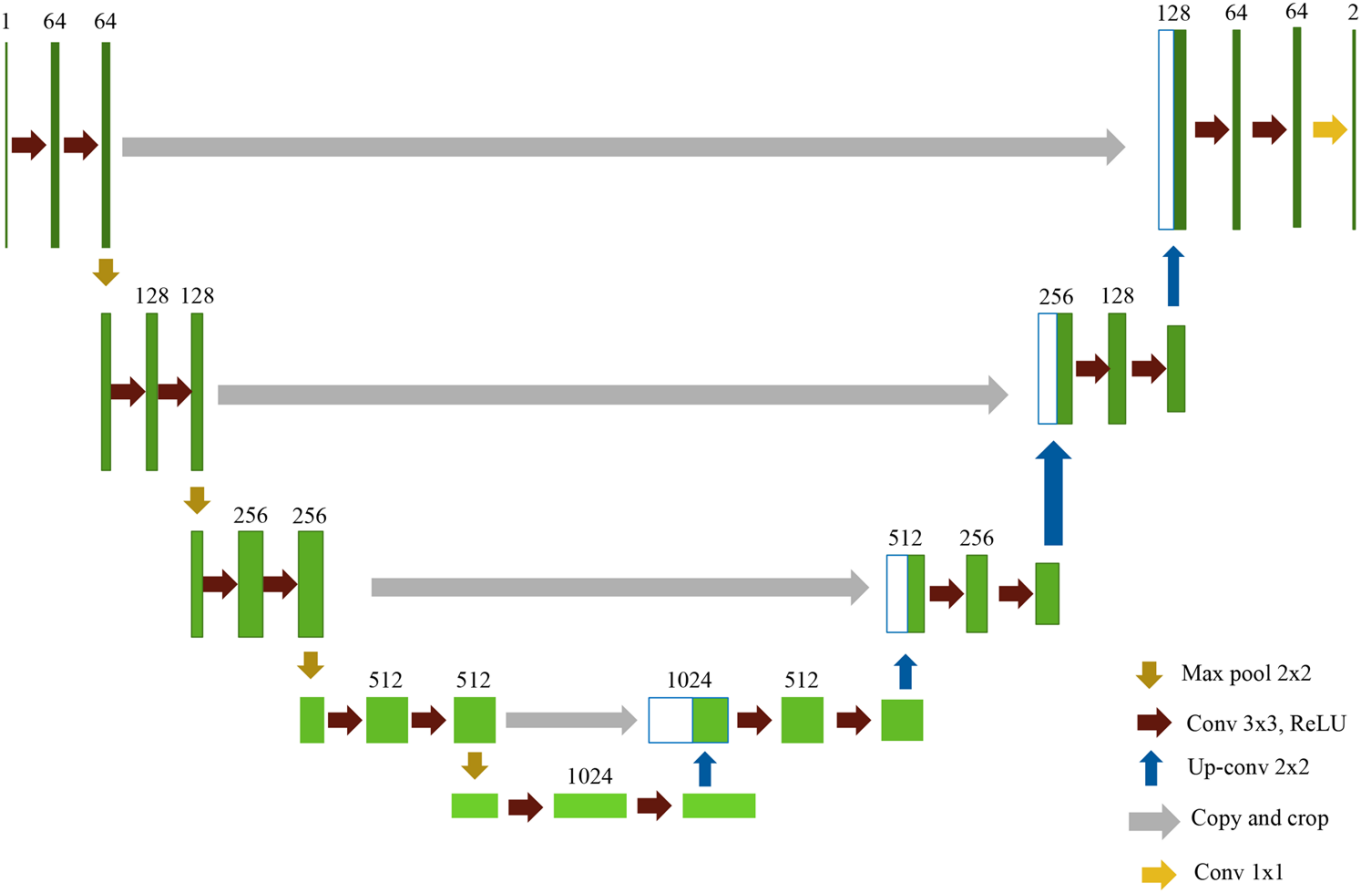
The structure of the U-Net model^39^.

Segmentation is one of the image processing methods, which separates the objects with different textures out of the background. Traditional segmentation approaches usually use morphological, thresholding, edge detection to divide the objects, which requires a lot of experiments and experts to perform the tasks. An alternative method, named U-Net model, which employs a convolutional neural network structure to predict the position of the texture, is the latest approach to improve the traditional ways for segmenting the texture^37–39^. This study utilized U-Net model to automatically learn and predict the tool wear areas. The shape of U-Net model is like the letter u, which is shown in Fig. 3. The architecture of U-Net model is composed of two parts: the left side and the right side. The left side can be treated as an encoder, and the right side is used as the decoder. The function of the encoder is used to extract the features and reduce the dimension, which is composed of convolution and pooling layers. And the decoder is used to reconstruct the smaller features into a new image with the same size of the input images. The main process of the decoder is performing the upsampling, which consists of unpooling and transpose convolution. The structure of U-Net model is similar to the autoencoder model, which also has the elements of the encoder and decoder. However, the bottleneck of the autoencoder is missing some features during the upsampling of reconstructing the images. U-Net model could overcome the drawbacks, which adds the connection between the encoder and decoder that the important information would not disappear in the process of reconstruction.

## Experiment and results

The proposed methods based on template matching, object detection, and segmentation methods are described and investigated in this section. First, the dataset used in this study are expressed in detail. Next, the following sections illustrate the experimental result of the template matching technique, YOLO v4, and segmentation models for detecting the tool flank wear areas. These models were trained in the GPU embedded with NVIDIA GeForce GTX 1050 Ti for computational acceleration. The deep learning framework Keras was used together with TensorFlow, a machine learning backend library.

### Dataset descriptions

**Figure 4:**
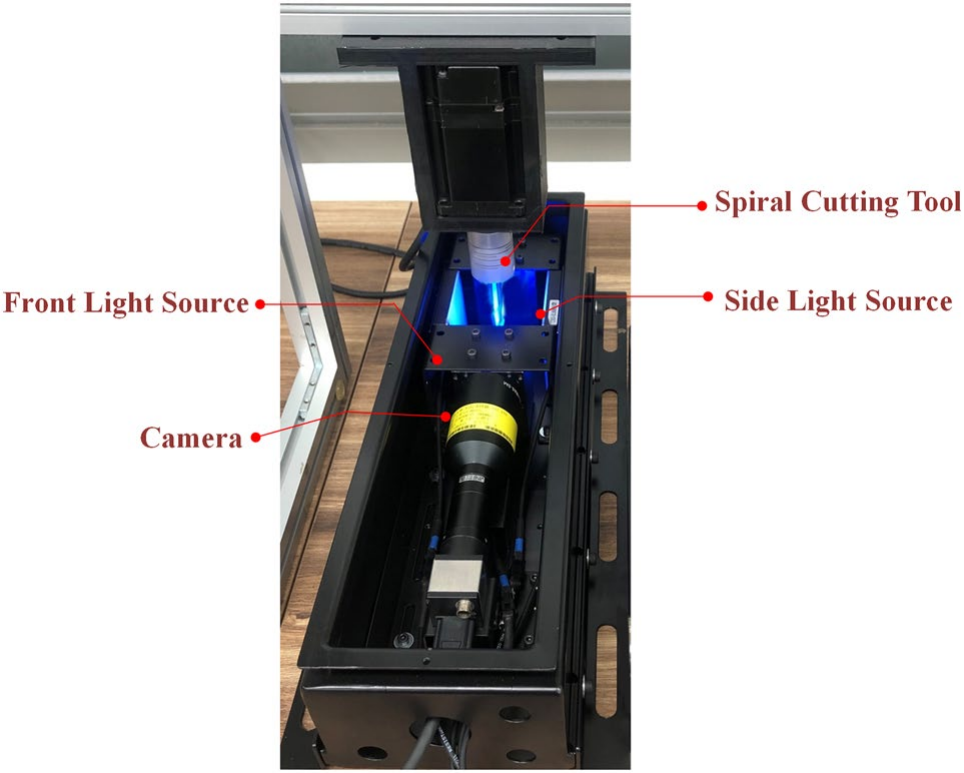
The developed optical instrument of tool wear monitoring system.

This study combines the template matching technique with deep learning methods to detect the tool wear defect of the spiral cutting tool. The dataset of the tool wear images are acquired from the developed optical instrument of the real-time tool wear monitoring system, which is shown as Fig. 4. This paper cooperated with Intelligent Mechatronic Control and Machinery lab in National Taiwan University department of mechanical engineering to collect the spiral tool wear images around 100 images for analysis. In order to capture the tool flank wear of the spiral tool, the location of the spiral cutting tool is perpendicular to the lens. The images of the tool flank wear of the spiral tool are captured approximately 210 images in 3 s. The spiral cutting tool is a NACHI square end mill with the material of super hard HSS-CO, which has two flutes for machining. The highest image quality of the tool flank wear images are captured with the iDS camera and MORITEX's lens. The front light source is used the blue coaxial light of CHD-FV40. And Backlighting of two sides of CHD-BL6060 is used as the side lighting source to create more light for capturing. In this experiment, the 100 spiral cutting tools datasets are expanded into panorama images for analyzing the tool wear areas. This study used 100 images of spiral cutting tools datasets for model learning and validation. And around 50 images are used to test the tool wear detection results of both YOLO v4 and segmentation models.

### Expanding the spiral cutting tool

**Figure 5:**
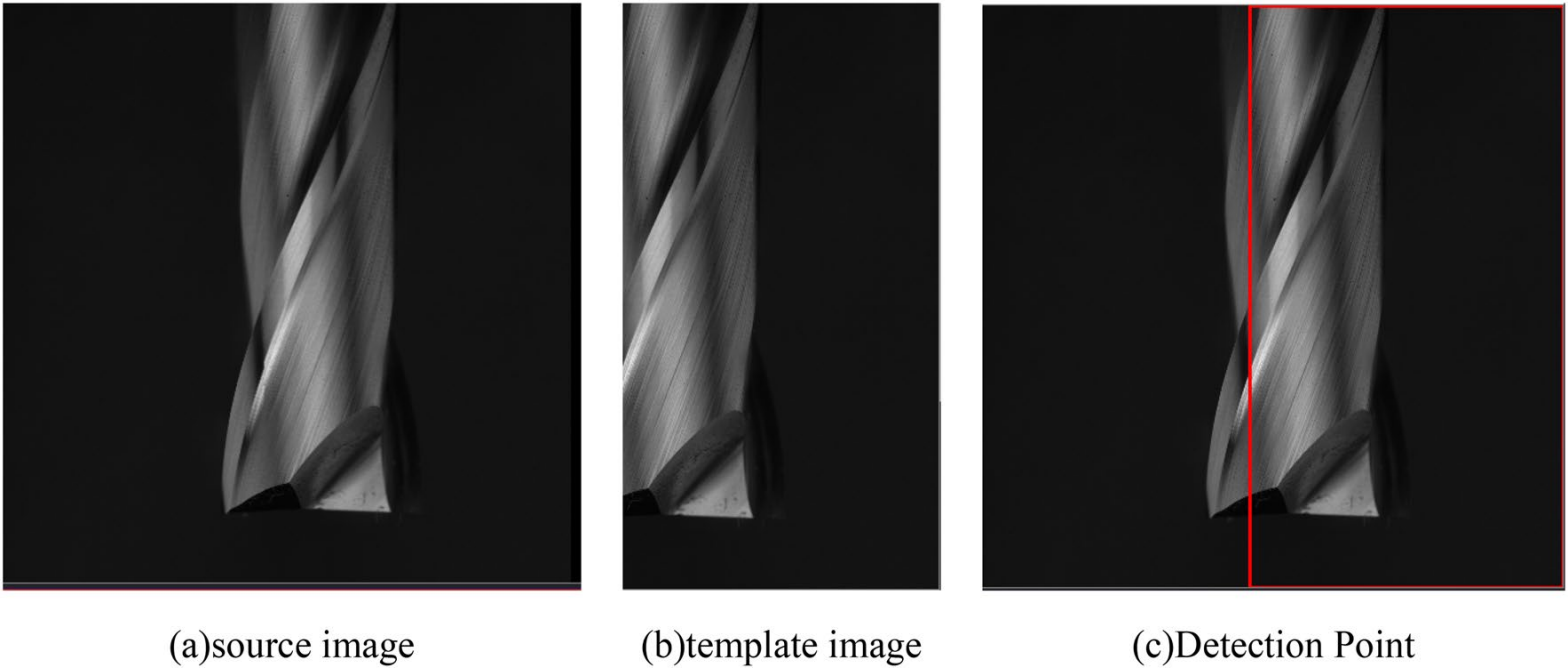
The schematic of the template matching method.

In this study, due to the characteristic of the curved surface of the spiral cutting tool, it is difficult to extract the wear region of the cutting tips. To address this issue, the cutting tool images were stitched and merged the curved surface of the spiral cutting tool into a panoramic image by using the template matching method. Template Matching is a method for searching and finding the location of a template image in a larger inspected image. The process of template matching is simple, which slides the template image over the input image and comparing the template with the patch of input image under the template image. The schematic of the template matching method is shown in Fig. 5. The template image indicates the image in which we expected to find a match region based on the source image. The template image would be compared to the source image based on the value of normalized cross correlation, which finds the similarity between the template image and the source image. The red bounding box means the high similar score area between the template and the source image. To obtain the stitched panoramic image of the two images, the location on the source will be replaced by the template image from the overlapped location. Table 1 illustrates the process of stitching several images into a panorama image. In order to merge into a panorama by using several tool flank images, the 360 degrees of the spiral cutting tool were captured into tool flank images per 1 degree. So, approximately 300 images of the spiral cutting tool were obtained for each tool. This study utilized approximately 500 spiral cutting tools for analysis. Table 2 shows the panoramic images of the spiral cutting tool with different machining parameters. As shown in Table 2, the result of the stitched image can be precisely aligned between the template image and the source image. The tool images with different machining parameters also can be precisely merged into a panorama image.

**Table 1:**
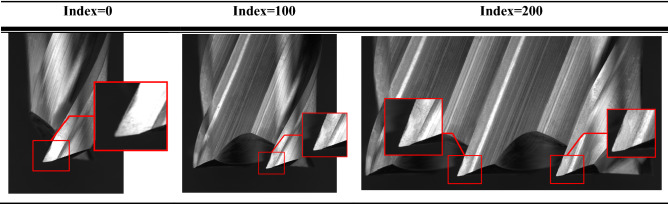
The process of stitching several images into panorama image, where the red box indicates the wear.

**Table 2:**
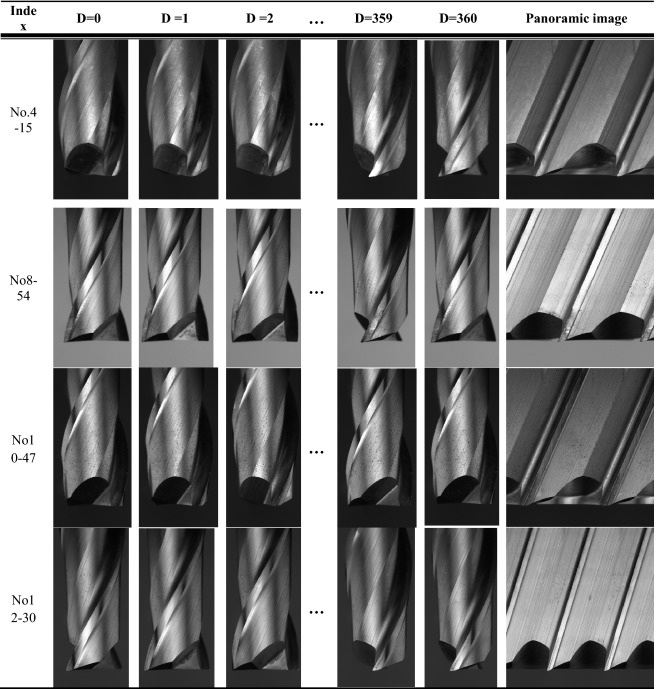
The expanded panorama images with different phases of each spiral cutting tool.

### Locating the cutting tips by using YOLOv4

**Figure 6:**
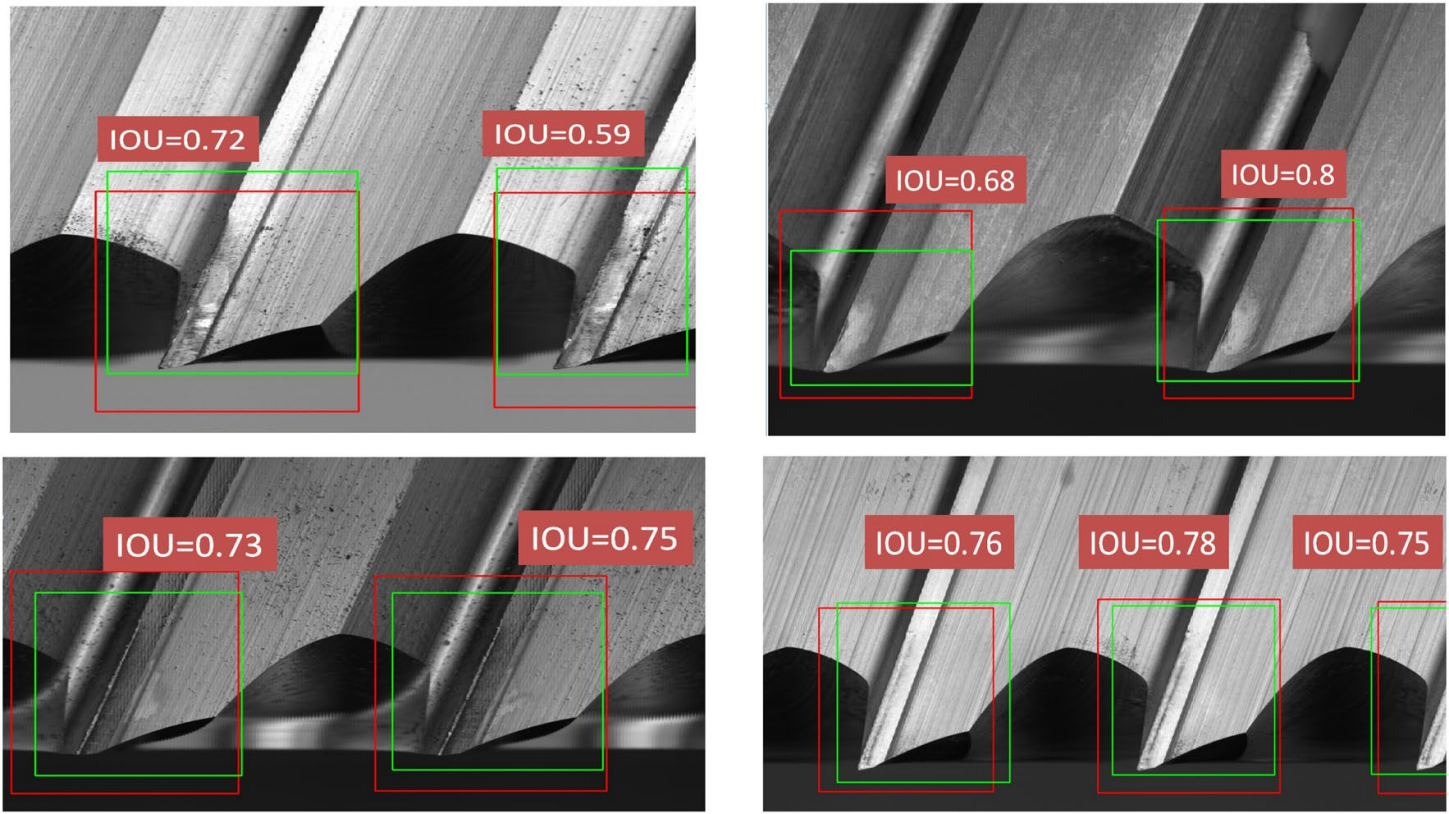
The results of detecting the cutting tips by using YOLO v4 model.

To enhance the performance of segmentation for tool wear areas, it is required to extract the boundary areas of cutting tips. Recently, several object detection methods are proposed and successfully detecting different classes of objects in an image. As the state-of-the-art object detector, YOLOv4 obtained better performance in detection speed (FPS) and detection accuracy (mAP) than all available methods due to the characteristic of CSPDarknet-53 combined with Spatial Pyramid Pooling in Deep Convolutional networks (SPPnet). After expanding into a panoramic image, the YOLOv4 model is utilized to locate the area of the cutting tip. For performing the object detection task, approximately 500 panoramic images were utilized for the training dataset and the testing dataset consisted of 50 images. During the network training, the parameters are set as follows: the batch size is 5 epochs, the number of steps per epoch is 200, the learning rate is 0.0001, and the loss function is selected binary cross entropy. The results of the testing images are shown in Fig. 6.

### Performance of the YOLO v4 model

To evaluate the performance of the YOLOv4 model, this paper utilized the unknown data as the testing dataset to assess the results of the training models. The standard statistical measures of Intersection over Union (IOU), Recall, Precision, and F1 score are usually employed to evaluate the location of the bounding box^40,41^. IOU is utilized to determine the similarity of the ground truth box with a predicted box of cutting tips region. The threshold of the IOU value was set to 0.5. If the IOU value is greater than 0.5, then the prediction of the cutting tip is truly positive and correct. Otherwise, it is considered a false positive. The definition of the IOU is given as Eq. (4). IOU is a ratio between the intersection with the union of the actual box and the predicted box. Moreover, precision, recall, and F1 score are also popular statistic indicators to evaluate the performance of the object detection models as well. The definition of precision, recall, and F1 score is provided in Eq. (5) to Eq. (7). The meaning of precision illustrates the detection ability with negative datasets. If the precision value is higher, distinguishing the negative dataset is stronger. The definition of Recall value is the ability of model detection with positive datasets. If the value of recall is higher, the model explanation with a positive dataset is better. The F1 score is integrated the mean of the recall and the precision value, reconciling the model with the precision and the recall value. The higher value of F1 score, the model is more robust. The detection results of the cutting tip with the indicators of precision, recall, and F1 score is given as Table 3. The results have been shown that the YOLO v4 model could achieve higher detection results on recognizing the boundary of cutting tips.

4 $${\text{IOU}}({\text{A}},{\text{B}}) = \frac{{{\text{A}} \cap {\text{B}}}}{{{\text{A}} \cup {\text{B}}}}$$

5 $${\text{Pecision}} = { }\frac{{{\text{TP}}}}{{{\text{TP}} + {\text{FP}}}}{ }$$

6 $$Recall = \frac{TP}{{TP + FN}}$$

7 $$F1 = { }2{*}\frac{Precision\,*\,Recall}{{Precision\, +\, Recall}}$$

**Table 3:** The detection results of the cutting tip with the indicators of precision, recall and F1 score.

mIOU	Recall	Precision	F1
0.67	0.92	0.87	0.89

In Eqs. (2) and (3), the TP, FP, and FN represent True Positive, False Positive, and False Negative, respectively. As the main task in this section, both precision and speed of object detection should be considered. The detection speed of the proposed system satisfies the real-time capability in terms of computational complexity. On the other hand, YOLOv4 is capable to identify the cutting tips with the different light sources. The results of detecting the cutting tips by using the YOLO v4 model are provided in Fig. 6.

### Detecting the tool wear areas using sematic segmentation

#### Definition of the tool wear

**Figure 7:**
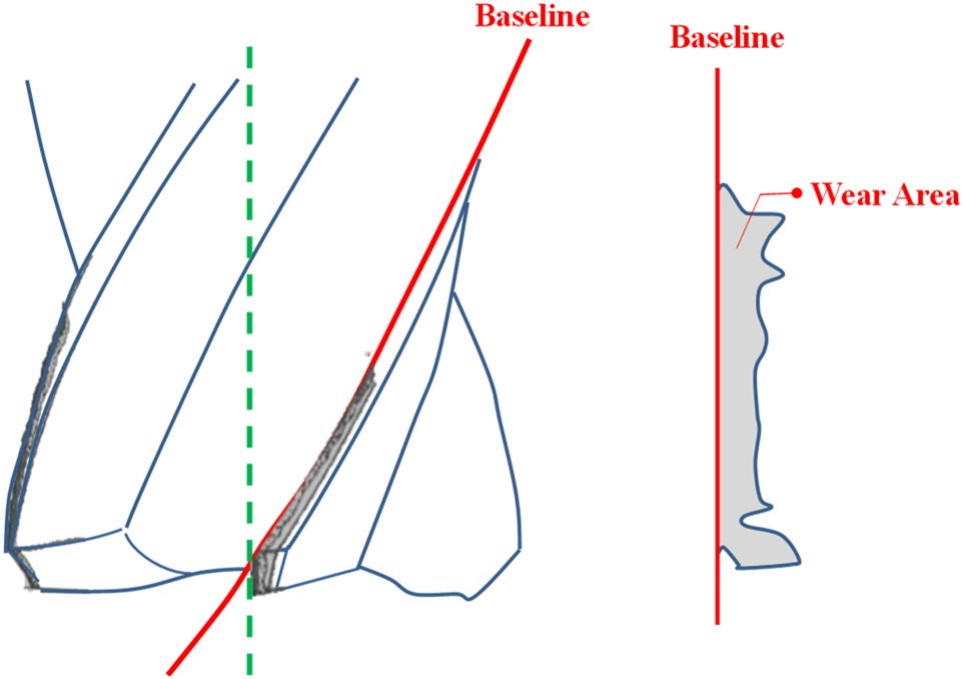
Definition of ISO tool wear^42^.

**Figure 8:**
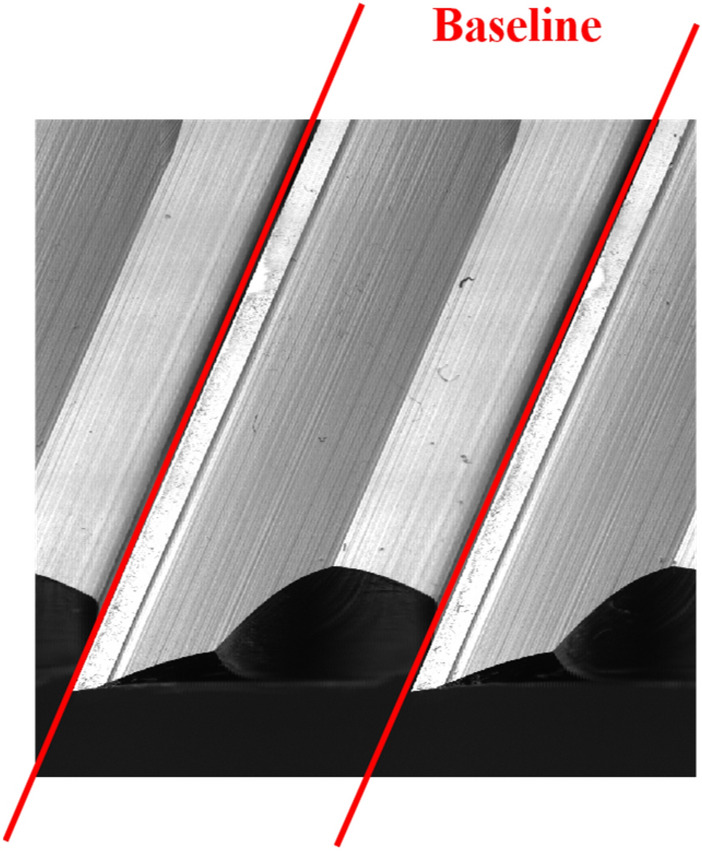
The expanded panorama image of spiral cutting tool.

It is vital to clearly understand the definition of tool wear. It helps us more clearly to develop the algorism for detecting the tool wear areas. According to ISO standards, the tool wear can be defined as Fig. 7. Tool wear causes changes in tool shape from the original shape. The extension of the baseline is used as the main reference line to detect the wear areas of the spiral cutting tool. The tool wear region is the deeper area of the right side based on the baseline. A red baseline is shown as Fig. 8. In order to obtain the baseline of the spiral cutting tool more effectively, this study employed the novel algorism of pattern matching method, which not only stitches the continuous images of the cutting tool images into a panorama image but also converting the spiral tool line into the straight line. The expanded panorama image of the spiral cutting tool is shown as Fig. 8. By the stitching method, it is able to capture the tool wear area without the step as selecting the specific area in traditional methods.

### Experiments result of U-Net model

U-Net model is mainly used to segment tool wear areas of the expanded panorama image in this article. This study selected approximately 200 cutting tip images from the tip detection phase as a training dataset. During the network training, the parameters were set as follows: the batch size was set to 10 epochs, the number of steps per epoch was 200, the learning rate was 0.0001, and the loss function was selected binary cross entropy to quantitatively evaluate the presented model. The indicators of intersection over union (IOU) and dice coefficient (DC) are the most generally utilized metrics in semantic segmentation^43^. The IOU and DC were used to assess the performance of segmented results. The definitions are provided in Eqs. (8) and (9). The predicted results of sematic segmentation models would be compared with ground truth (GT).

8 $${\text{IOU}} = { }\frac{{\left| {Predict \cap GT} \right|}}{{\left| {Predict \cup GT} \right|}}{ }$$

9 $${\text{Dice Coefficient}} = { }2{*}\frac{{\left| {Predict \cap GT} \right|}}{{\left| {Predict} \right| + \left| {GT} \right|}}{ }$$

In order to evaluate the detection results of the segmentation model, the testing samples are utilized to verify the performance of the U-Net segmentation model. Moreover, this study also compares two segmentation models, Segnet and Autoencoder. All datasets were tested under the same circumstances of the model parameters. The metrics of Dice Coefficient and IOU value are used to assess the three models, namely, U-Net, Segnet and Autoencoder, are shown in Table 4. The results show that the average IOU of Segnet, Autoencoder, and U-Net models are 0.37, 0.23, and 0.47, respectively. U-Net model obtains the best average IOU value, which represents the prediction tool wear areas almost enclosed the ground truth. The dice coefficient of the U-Net model also achieves the highest value among the segmentation models. It can also reveal that the performance of the U-Net model is superior than Segnet and Autoencdoer models. Table 5 shows the detail of the three models results. Moreover, Table 5 also shows the detection efficiency in real-time, which is called frame per second (FPS). The results reveal that due to the complexity of the U-Net model, resulting in the slower detection speed.

**Table 4:**
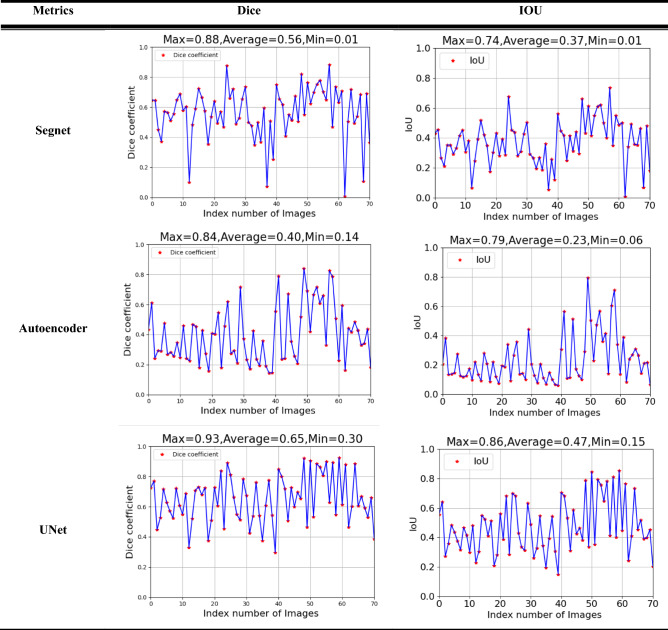
The evaluation of Segnet, Autoencoder, and U-Net models.

**Table 5:** Results of three models.

Model	Dice min/mean/max	IOU min/mean/max	FPS (70frame)
U-Net	0.3/0.65/0.93	0.15/0.47/0.86	19
Autoencoder	0.14/0.4/0.84	0.06/0.23/0.79	25
Segnet	0.01/0.56/0.88	0.01/0.37/0.74	13

Table 6 shows the detection results by using the U-Net model. The overview of the images on No. 1 to No. 4 presented that even the different angles of cutting tips, the tool wear areas can be effectively extracted by using the U-Net model. Moreover, the predicted mask of the tool wear area is almost fitted with the ground truth mask. The comparison of detecting the tool wear areas by using three models are shown as Table 7. Among three semantic segmentation models, the U-Net model can precisely extract the tool wear areas from the cutting tips regions. The detection results of the U-Net model are superior than the other two models.

**Table 6:**
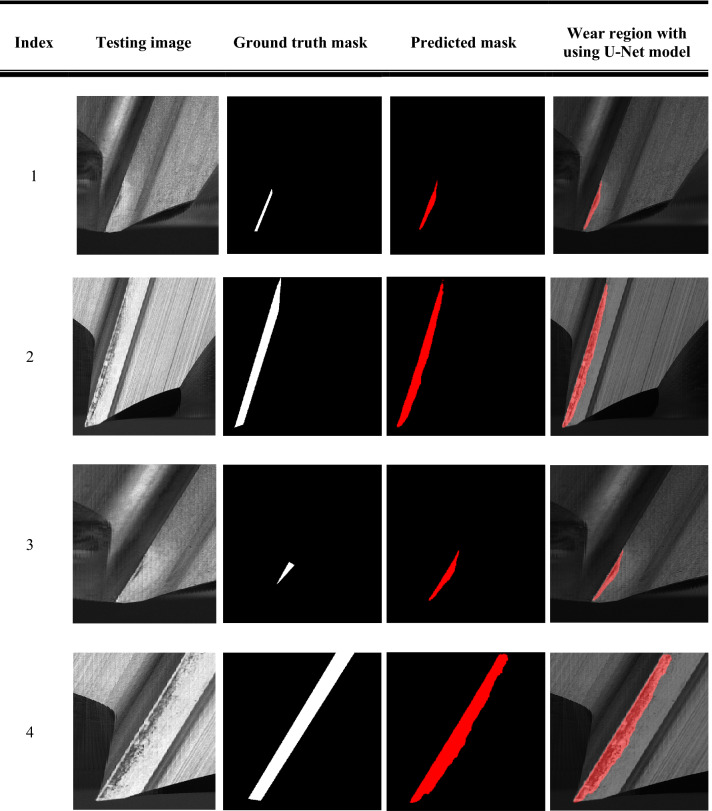
The detection results by using U-Net model.

**Table 7:**
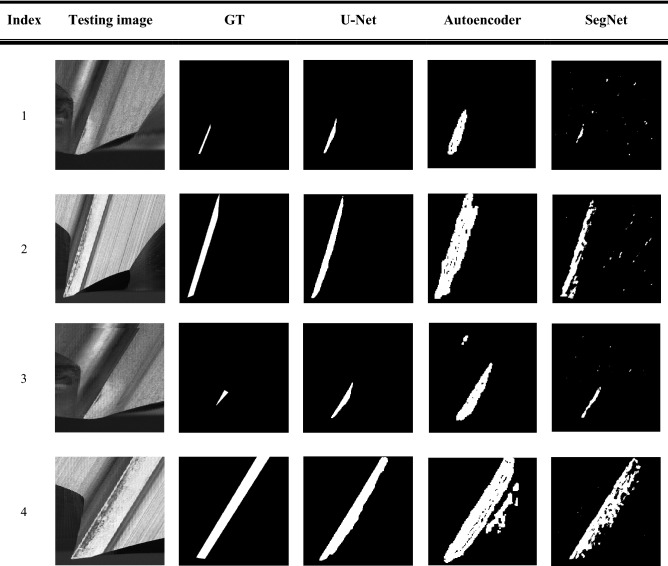
The comparison of detecting the tool wear areas by using three models.

**Figure 9:**
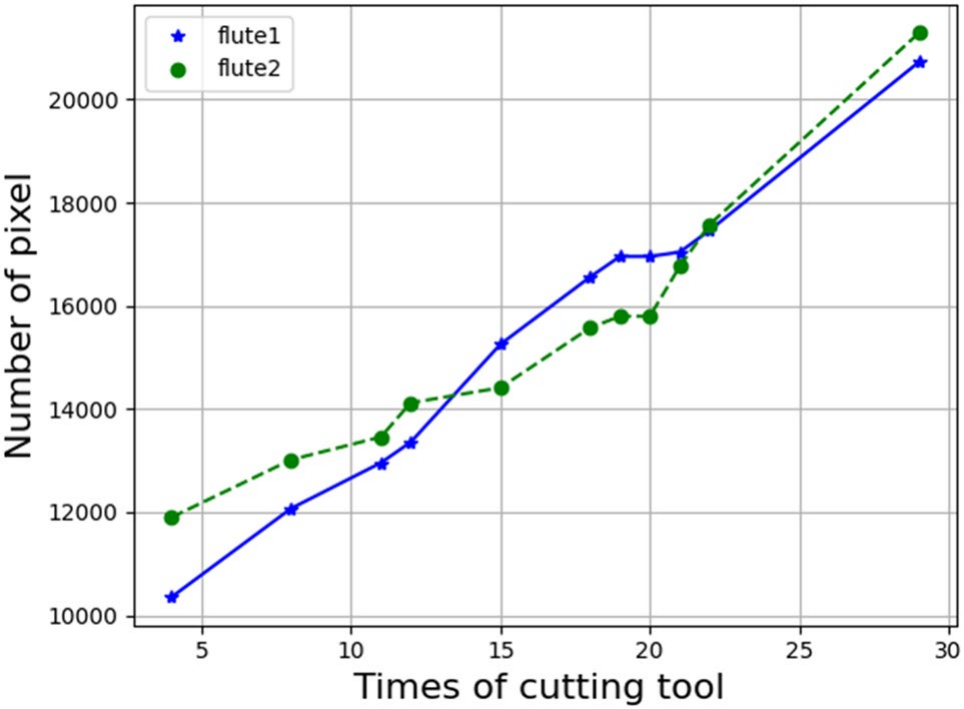
The experimented trend of the cutting tool wear status.

To explore the relationship between the machining times and the tool wear areas, this paper selected a cutting tool as a sample to investigate the wear condition of the machining process. According to the above prediction results of sematic segmentation models, the U-Net model achieves the higher prediction output. This paper further analyzes the wear areas of the cutting tool processing process based on the prediction results of the U-Net model. The experimented trend of the cutting tool wear status is shown as Fig. 9. This paper analyzed each machining process results of tool wear areas from unprocessed to 30 times of processing. Because this paper used the spiral cutting tool with two flutes that two curves are shown in the Fig. 9, which represents the first flute and the second flute. The unit of the wear areas is a pixel, and the pixel size is 11.5 um. It can be observed that with the increasing of the machining times, the tool wear has become more severe. By the tool wear curve of the machining process, users can not only realize the wear status of the cutting tool but also determine the times of the tool change depend on the curve of the tool wear, which can prevent the cutting tool being worn severely to ensure the quality of the machined products.

## Discussion

It is important to realize the tool wear condition during the machining process. The spiral cutting tool is a common machining tool. The characteristic of the spiral cutting tool has a curved surface, resulting the difficulty detecting the wear region of the cutting tips. To achieve comprehensively automatic detecting the tool wear area of the spiral cutting tool, the tool wears detecting system is proposed in this study. The image stitching of template matching algorism is used to stitch and merge the several curved surface images of the spiral cutting tool into a panoramic image. By using the image stitching method, it can also convert the spiral cutting line into a straight line to detect the region the cutting tool more flexible. However, the panorama image of the spiral cutting tool is larger, the YOLO v4 model is employed in this article to automatically detect the range of cutting tips. To detect the tool wear areas, image semantic segmentation is utilized in this study. Three semantic segmentation models, namely, Segnet, Autoencoder, and U-Net model, are compared to explore the performance of segmentation. Among the three semantic segmentation models, U-Net achieves the best average IOU and dice coefficient score by extracting the wear areas of the panoramic cutting tool image. The experiment results reveal that the U-Net model has the characteristic of concatenating the feature maps from low to high level, which can achieve high identification results. Furthermore, this study utilized the U-Net model to predict the wear areas of the spiral cutting tool during the machining process, and draw it into the trend figure. The experimented trend of the tool wear areas can provide users with detailed information about the tool wear status. It can prevent the cutting tool before being worn severely to change the cutting tools in advance and avoiding serious impact on the machining quality. The study presented an effective detection method, which combines the template matching with a deep learning technique to recognize the tool wear area of the spiral cutting tool. The proposed methods are able to capture the tool wear area without the step as choosing the specific area in traditional approaches, which can achieve the on-site usage requirements. The developed system can reduce the frequency of changing the cutting tool to check the tool wear status, and improve the machining efficiency.
