# Publisher Correction: Integrating object detection and image segmentation for detecting the tool wear area on stitched image

**DOI:** 10.1038/s41598-021-01172-y

**Published:** 2021-11-01

**Authors:** Wan-Ju Lin, Jian-Wen Chen, Jian-Ping Jhuang, Meng-Shiun Tsai, Che-Lun Hung, Kuan-Ming Li, Hong-Tsu Young

**Affiliations:** 1 Department of Mechanical Engineering, National Taiwan University, Taipei, 106319 Taiwan; 2 Institute of Biomedical Informatics, National Yang Ming Chiao Tung University, Taipei, 11221 Taiwan; 3 Department of Computer Science, National Tsing Hua University, Hsinchu, 300044 Taiwan; 4 Department of Computer Science and Communication Engineering, Providence University, Taichung City, 43301 Taiwan

Correction to: *Scientific Reports* 10.1038/s41598-021-97610-y, published online 7 October 2021

Hong-Tsu Young was omitted from the author list in the original version of this Article.

The original Article has been corrected.

